# Facing a Health Threat in a Complex Information Environment: A National Representative Survey Examining American Adults’ Behavioral Responses to the 2009/2010 A(H1N1) Pandemic

**DOI:** 10.1177/1090198117708011

**Published:** 2018-02

**Authors:** Leesa Lin, Rachel F. McCloud, Minsoo Jung, Kasisomayajula Viswanath

**Affiliations:** 1 Harvard T. H. Chan School of Public Health, Boston, MA, USA; 2 Dana-Farber Cancer Institute, Boston, MA, USA; 3 Dongduk Women’s University, Seoul, South Korea

**Keywords:** emergency risk communication, H1N1, health behavior, nonpharmaceutical interventions, pandemic, vaccination

## Abstract

*Background*. Recent A(H1N1) studies suggest that intrapersonal and interpersonal factors may exert influence on people’s preventive behaviors for avoiding the flu during pandemics. *Aims*. Nonpharmaceutical interventions (NPIs) and vaccinations play key roles in containing disease transmission during a pandemic. We examined how intrapersonal and interpersonal factors influenced adoption of NPIs and vaccine uptake during the A(H1N1) pandemic of 2009. *Method*. The data come from a nationally representative sample survey of 1,569 American adults. Hierarchical multivariable logistic regression was conducted to investigate the association between socioeconomic position, concern and knowledge about the threat, social networks for health advice or health care seeking, health consultations with doctors, and NPIs (including individual’s social distancing behaviors and hygiene practices) and vaccine acceptance. *Results*. People with higher scores on health-related social networks, more knowledge or concern about A(H1N1), and those who have consulted their doctor were more likely than others to adopt NPIs. There was a significant association between being concerned about A(H1N1), having consulted a doctor, and seeking a vaccine. *Conclusions*. These findings suggest that interpersonal communication factors, such as health-related social networks and consultations with doctors, and intrapersonal factors, such as concern and knowledge, play a critical role in NPIs and vaccine uptake during pandemics and offer avenues for intervention.

High levels of uncertainty regarding an epidemic’s characteristics, transmission prevention, and treatment are common during the early stages of disease outbreaks, posing numerous challenges to public health communication, such as knowing what to communicate about risks and preventative measures, and how to communicate such information, while simultaneously managing public fear. As the epidemic evolves and a vaccine becomes available, public health communications about the epidemic shift to promoting vaccination, addressing potential concerns with side effects, and countering negative messages (Galarce, Minsky, & Viswanath, 2011; Henrich & Holmes, 2011).

Nonpharmaceutical interventions (NPIs), one of the 15 Public Health Preparedness Capabilities identified by the U.S. Centers for Disease Control and Prevention (CDC), are nonmedical strategies for disease, injury, and exposure control, and include social distancing, hygiene, and other precautionary protective behaviors (CDC, 2011). By reducing contact rates between susceptible individuals and possibly infected individuals, social distancing practices—reducing human-to-human contact and avoiding public spaces—are cost-effective strategies that may greatly reduce disease transmission (Perlroth et al., 2010). Frequent hand-washing (Godoy et al., 2012; Suess et al., 2012) and facemasks (Suess et al., 2012) can also significantly reduce pandemic flu cases during an outbreak. Until vaccines or other medical measures become available, high levels of compliance with NPIs are needed to control disease transmission and mitigate harm (CDC, 2007; Halloran et al., 2008; World Health Organization, 2006). The role of public communication and other factors in influencing the uptake of NPIs and compliance with recommended practices is critical and merits further study. The observation of suboptimal vaccination rates, as seen during seasonal flu and the 2009/2010 Influenza A(H1N1) outbreaks, demonstrate the importance of promoting NPIs uptake (CDC, 2010a, 2010b), even after vaccines become available.

The 2009/2010 A(H1N1) pandemic provided an optimal opportunity to observe the barriers and facilitators to NPIs-related behaviors and vaccine uptake. For example, in a nationally representative U.S. sample, 72% reported more frequent hand-washing or hand sanitizer use during the 2009 pandemic, 28% avoided places where people gather, 23% avoided bus or plane travel, and 16% took public transportation less frequently (SteelFisher et al., 2012).

Studies conducted contemporaneously to the 2009/2010 A(H1N1) pandemic suggest that multiple factors at the individual level influence the adoption or negligence of preventive behaviors, including intrapersonal traits (e.g., pandemic-related concern), interpersonal communications (e.g., influences from social networks and provider recommendations), and knowledge (Jung, Lin, & Viswanath, 2013; Kumar et al., 2012; Lin, Jung, McCloud, & Viswanath, 2014).

Socioeconomic position (SEP)—measured by income, education, and employment, represents a similarly critical factor. For example, A(H1N1)-related knowledge is associated with higher adoption of NPIs and vaccines but is lower among low-SEP individuals (Lin, Jung, et al., 2014; Tooher, Collins, Street, Braunack-Mayer, & Marshall, 2013). Vaccine uptake was significantly associated with income, education, vaccine access, and personal cost (Galarce et al., 2011; Plough, Bristow, Fielding, Caldwell, & Khan, 2011). Other individual factors, such as pandemic concern, were linked to increased vaccine and NPIs uptake, including avoiding crowds (Liao, Cowling, Lam, Ng, & Fielding, 2014; Serino et al., 2011) and frequent hand-washing (Plough et al., 2011; Rubin, Potts, & Michie, 2010; Serino et al., 2011).

At the interpersonal level, social networks may influence pandemic-related vaccination and information gathering. An individual’s decision to receive pandemic flu vaccination may be guided by communications in their health-related social networks in which people discuss health issues, seek advice, or get rides to the doctor (Nyhan, Reifler, & Richey, 2012), especially among individuals with higher tendencies toward social conformity (Xia & Liu, 2013) and those who believe others want them to get vaccinated (Bish, Yardley, Nicoll, & Michie, 2011; Kumar et al., 2012). Although relatively less research focuses on how social networks influence NPIs, information seeking during pandemics has been found to largely originate within social networks. Social networks spread interest, and individuals may imitate their peers’ Internet search behavior for pandemic-related information (Bentley & Ormerod, 2010). Thus, the interpersonal context of pandemic-related communication may significantly influence preventive behaviors (Jung et al., 2013; Prematunge et al., 2012). Receiving vaccination advice from doctors strongly predicted vaccine acceptance (Bish et al., 2011; Brien, Kwong, & Buckeridge, 2012; Davila-Payan, Swann, & Wortley, 2014; Prematunge et al., 2012; Rodas et al., 2012).

Research has identified barriers and facilitators of NPIs uptake, but most research examined only one set of factors at a time when, realistically, outcomes are likely influenced by multiple sets of factors. We were interested in how individual *and* social determinants combine to affect individual behaviors. Drawing from a variety of theories in health behavior and social determinants literature, we were interested what factors potentially influence NPIs uptake and vaccination-related decisions. For example, theories such as health belief model argued for the importance of concern and risk perception in influencing health behaviors (Skinner, Tiro, & Champion, 2015). The structural influence model of communication (SIM) highlights the importance of social determinants, such as SEP, race, and ethnicity on interpersonal communications and information seeking (among others), and how these may influence health outcomes (Viswanath, Ramanadhan, & Kontos, 2007). The extensive literature on social determinants documented how factors, such as SEP, race, ethnicity and geography among others, either individually or with other factors, drive health behaviors. Thus, from a strategic communication perspective, we were interested in factors communications can modify or address, such as interpersonal social networks, through which information spreads to change pandemic-related concern (perceptions) and knowledge and, eventually, behavior. Specifically, this study investigated how SEP, health-related social networks, including consultations with health care providers and individuals’ affective (concern) and cognitive (knowledge) factors, contribute to public health emergency preparedness (PHEP) behavioral outcomes, such as NPIs and vaccine acceptance, during pandemics.

## Method

### Sample

Data for this study come from a survey of 1,569 respondents, drawn from a nationally representative sample of U.S. adults aged 18 years and older, collected in late February and early March 2010. Respondents were drawn from the Knowledge Networks’ KnowledgePanel^©^, recruited using a dual sampling frame, a combination of Random Digital Dial and Address-Based Sampling, allowing for sampling individuals without telephone landlines. Households were provided Internet access and necessary hardware, if needed. Poststratification weights were used to adjust for noncoverage and nonresponder biases. Demographic and geographic distributions for the population aged 18 years and above, from the most recent Current Population Survey (U.S. Census Bureau, 2009) and the 2006 Pew Hispanic Center Survey of Latinos (Suro & Escobar, 2006), are used as benchmarks in this adjustment. Poststratification weighting included gender, age, race/ethnicity, education, census region, urbanicity, Internet access, and primary spoken language (Dennis, 2010; GfK, 2011). For this study’s purposes, participants from minority ethnic/racial groups and those living under the Federal Poverty Level were oversampled—allowing overall survey results to represent the national and oversampled populations. The total percentage of missing values were very low (<1.3%, *N* < 20)—these values were missing completely at random and therefore were excluded from final analyses according to the diagnostic results.

### Survey Questions

Survey questions used to gather data for this article were systematically developed, starting with a literature review (Galarce et al., 2011; Lin, Savoia, Agboola, & Viswanath, 2014; Savoia, Lin, & Viswanath, 2013) followed by five focus groups with participants from diverse ethnic/racial and socioeconomic backgrounds in Massachusetts (McCauley, Minsky, & Viswanath, 2013). Key themes were related to preventive behavior, health communication, and H1N1 knowledge. Themes were combined with items adapted from the Health Information National Trends Survey (Cantor, Crystal-Mansour, Davis, Dipko, & Sigman, 2007) and Harvard Opinion Research Program H1N1 Surveys (Blendon et al., 2009-2010). The survey was finalized after cognitive interviews with potential respondents to evaluate potential sources of response error and improve the instrument.

### Variables

#### Independent Variables

This study included five sets of individual and social determinants derived from the literatures on SIM, health behaviors, and social determinants as independent variables:

##### Individual factors

1. *SEP*: household income, educational attainment, and employment status.
2. *A(H1N1)-related health concern* refers to the extent people were concerned about A(H1N1) affecting themselves or their immediate family’s health. Participants were asked to respond to the following: “Are you concerned that you may get sick from A(H1N1) during the next 12 months, or are you not you concerned about that?” and “Are you concerned that someone in your immediate family may get sick from A(H1N1) during the next 12 months, or are you not you concerned about that?” Respondents answered on a 0 (*not at all concerned*) to 10 (*very concerned*) scale. Responses were combined into one score (ranging from 0-20) and recoded as one categorical variable: *not really concerned* (score = 0-4), *little concerned* (score = 5-10), *somewhat concerned* (score = 11-15), and *very concerned* (score = 16-20).
3. *Assessments of A(H1N1)-related knowledge*: To determine respondents’ levels of knowledge about A(H1N1) transmission, they were asked: “To the best of your knowledge, how can someone get H1N1?” Respondents obtained a score of 0, 1, or 2. To account for randomly guessed responses, correct answers were discounted when respondents selected incorrect answers. One point was received for each of the following correct options chosen: “From touching objects (i.e., glass) recently touched by someone with flu” and “From being in *close* contact with someone who has H1N1 (within arms-length of someone),” and if none of the following were chosen: *from eating pork*, *from coming in contact with pigs*, and *none of the above.* Knowledge assessment scores were adapted from previously-tested measures (Lin, Jung, et al., 2014; Savoia, Testa, & Viswanath, 2012).

##### Interpersonal communication factors

1. *Health-related social networks* were assessed by three statements that participants responded to with *yes* or *no*: (a) *“*Do you often discuss health related issues with friends and family?” (b) “In addition to your health care provider, do you have close friends or family members who can provide you with accurate advice on health?” and (c) “Do you have any close friends or family members you can rely on in case you need a ride to visit the doctor?” (0 = no and 1 = yes) Responses were combined into a score ranging from 0 (*no health-related networks*) to 3 (*strong health-related networks*).
2. *Health information seeking*: capturing individuals’ purposive seeking of A(H1N1)-related information and advice from doctors, items asked participants whether they had “Talked with your doctor about health issues related to H1N1” (“yes” = *seekers*, “no” = *nonseekers*).

#### Dependent Variables

Uptake of precautionary measures recommended by the CDC were used as outcome variables measuring participant’s behavioral responses to the A(H1N1) pandemic.

1. *Social distancing practices* included: “avoided places where many people are gathered together, like sporting events, malls, or public transportation,” “reduced human contact with people outside of your immediate family such as signs of affection (hug/kiss), shaking hands or sign of peace during worship,” and “stayed home.”
2. *Hygienic practices* included: “washed your hands or used hand sanitizer more frequently,” “worn a face mask,” and “began coughing with your mouth covered.”
3. *Vaccine acceptance* was measured by whether the participants “sought out a vaccine for you or your loved ones.”

The above variables were dichotomously coded as “1” for items checked and “0” for items unchecked.

#### Potential Covariates

The following confounders were controlled in the analyses, including: *age, sex*, and *race/ethnicity.*

### Analysis

Descriptive analyses, expressed as weighted frequencies and percentages, were performed to account for complex sampling design and to ensure estimates would be nationally representative. We applied binary logistic regression to test for significant bivariate associations between predictors and outcome variables. Based on literatures on SIM, health behaviors, and social determinants, we conducted hierarchical multivariate logistic regression analyses and tested behavioral responses to A(H1N1)―including social distancing behaviors, hygiene practices, and vaccine acceptance―as major outcomes of interest. Independent variables included *SEP* (Tier 1); *health-related social network and health consultations with doctors* (Tier 2), and *knowledge about A(H1N1) virus transmission and A(H1N1)-related concern* (Tier 3). All analyses used a conventional significance level, *p* < .05. All analyses were conducted using the statistical package STATA v11.0 (StataCorp, 2013).

## Result

### Sample Demographics

As Table 1 demonstrates, approximately 50% of the weighted sample population was under 44 years of age, 44% had at most a high school education and 14% earned under $15,000 annually. Sixty-eight percent were White, 11% non-Hispanic Black, 14% Hispanic, and 7% Other Race. Hygiene practices were followed more than other NPIs in response to A(H1N1): 66% of respondents reported washing hands or using hand sanitizer more frequently than before the A(H1N1) pandemic and 20% of respondents adopted social distancing practices. Regarding medical services utilization, 23% of respondents sought vaccination for themselves or loved ones and 14% asked doctors for consultations regarding A(H1N1).

**Table 1.** Distribution of Sample Characteristics.

Variable	Frequency (weighted percentage)
Demographic information	
Sex	
Male	681 (49)
Female	888 (51)
Age (years)	
18-24	154 (11)
25-34	343 (19)
35-44	335 (19)
45-54	293 (17)
55+	444 (34)
Race/ethnicity	
Non-Hispanic White	625 (68)
Non-Hispanic Black	134 (11)
Hispanic	728 (14)
Other	82 (7)
Social economic positions	
Education	
Bachelor’s degree or higher	283 (28)
Some college	467 (29)
High school	455 (30)
Less than high school	364 (14)
Household income	
≤$14,999	533 (14)
$15,000-$34,999	393 (21)
$35,000-$59,999	269 (23)
≥$60,000	374 (42)
Employment status	
Employed	735 (55)
Unemployed	451 (21)
Retired	383 (24)
Health behaviors	
Social distancing	
Avoided places where many people are gathered together	417 (20)
Reduced human contact with people outside of your immediate family	383 (21)
Stayed home	251 (10)
Hygiene practices	
Washed your hands or used hand sanitizer more frequently	1061 (66)
Worn a face mask	97 (5)
Began coughing with your mouth covered	607 (32)
Vaccine uptake	
Sought out a vaccine for you or your loved ones	410 (23)

### Hygiene Practices (Table 2)

Our analyses showed adopting hygiene practices is significantly and positively associated with A(H1N1)-related concern and knowledge, having health-related social networks, and consulting with doctors. Particularly, those experiencing A(H1N1)-related concern are at least three times more likely than people with no concern to practice hand hygiene. Those concerned about A(H1N1) are also more likely to wear a face mask. A similar trend is observed in health-related social networks. The higher score on health-related social network, the more likely people are to wash hands and/or use hand sanitizer frequently. Additionally, covering one’s mouth from a when coughing, an NPI, was more commonly observed among those with highest level of health-related social networks (adjusted odds ratio [AOR] = 2.70), who were very concerned about A(H1N1) (AOR = 2.53), were most knowledgeable about A(H1N1) transmission (AOR = 2.11), and who talked with doctors about A(H1N1) related health issues (AOR = 3.39).

**Table 2.** Hierarchical Logistic Regression Analyses Examining the Association Between *Hygiene Practices* and (a) Socioeconomic Position (SEP); (b) SEP and Interpersonal Networks: Health-Related Social Networks and Health Information-Seeking Behaviors; (c) SEP, Interpersonal Networks, and Intrapersonal Factors: A(H1N1)-Related Concern and Knowledge.

*Worn a face mask*			Tier 1: SEP			Tier 2: SEP and interpersonal networks			Tier 3: SEP, interpersonal networks, and intrapersonal factors		
Factors	Items	Percentage	aOR	*p*	95% CI	aOR	*p*	95% CI	aOR	*p*	95% CI
Household income	≤$14,999	14	Reference			Reference			Reference		
	$15,000-34,999	21	0.80	.69	[0.27, 2.36]	0.76	.60	[0.27, 2.11]	0.62	.37	[0.22, 1.75]
	$35,000-59,999	23	0.52	.19	[0.20, 1.37]	0.62	.32	[0.24, 1.61]	0.69	.47	[0.25, 1.91]
	≥$60,000	42	0.19	**<.005**	[0.06, 0.59]	0.19	**<.01**	[0.06, 0.61]	0.22	**<.005**	[0.08, 0.60]
Education	Bachelor’s degree or higher	28	Reference			Reference			Reference		
	Some college	29	0.60	.32	[0.22, 1.65]	0.71	.51	[0.25, 1.96]	0.48	.18	[0.16, 1.41]
	High school	30	1.23	.70	[0.43, 3.53]	1.33	.60	[0.45, 3.90]	1.19	.78	[0.37, 3.84]
	Less than high school	14	2.49	.10	[0.84, 7.37]	2.65	.07	[0.94, 7.49]	2.48	.09	[0.86, 7.17]
Employment status	Employed	55	Reference			Reference			Reference		
	Unemployed	21	0.60	.37	[0.20, 1.82]	0.64	.43	[0.22, 1.90]	0.56	.23	[0.21, 1.44]
	Retired/disabled	24	0.65	.34	[0.27, 1.58]	0.67	.35	[0.29, 1.55]	0.56	.18	[0.24, 1.32]
Health-related social networks	Score of 0 (*no health-related networks*)	7				Reference			Reference		
	Score of 1	21				0.53	.25	[0.17, 1.58]	0.37	.11	[0.11, 1.24]
	Score of 2	32				1.11	.85	[0.39, 3.17]	1.07	.90	[0.34, 3.40]
	Score of 3 (*strong health-related networks*)	41				0.91	.85	[0.34, 2.43]	0.77	.62	[0.27, 2.19]
Talked with doctors about health issues related to H1N1	No	86				Reference			Reference		
	Yes	14				3.17	**<.01**	[1.41, 7.14]	2.49	**.03**	[1.12, 5.55]
Concerned about self or someone in your immediate family getting sick from H1N1 during the next year	Not much concerned	38							Reference		
	Little concerned	38							5.31	<.005	[1.88, 14.97]
	Somewhat concerned	16							3.57	.01	[1.29, 9.86]
	Very concerned	8							12.13	<.001	[3.45, 42.66]
Knowledge about A(H1N1) virus transmission	Score of 0 (no correct answer)	10							Reference		
	Score of 1 (one correct answer)	45							1.49	0.35	[0.64, 3.49]
	Score of 2 (two correct answers)	44							0.90	0.80	[0.38, 2.09]
*Washed your hands or used hand sanitizer more frequently*			Tier 1: SEP			Tier 2: SEP and interpersonal networks			Tier 3: SEP, interpersonal networks, and intrapersonal factors		
Factors	Items	Percentage	aOR	*p*	95% CI	aOR	*p*	95% CI	aOR	*p*	95% CI
Household income	≤$14,999	14	Reference			Reference			Reference		
	$15,000-34,999	21	1.27	.38	[0.74, 2.18]	1.02	.94	[0.60, 1.74]	0.96	.88	[0.54, 1.69]
	$35,000-59,999	23	0.96	.87	[0.56, 1.64]	0.98	.94	[0.57, 1.67]	1.02	.95	[0.59, 1.77]
	≥$60,000	42	1.36	.24	[0.82, 2.28]	1.22	.45	[0.73, 2.07]	1.26	.40	[0.74, 2.15]
Education	Bachelor’s degree or higher	28	Reference			Reference			Reference		
	Some college	29	0.92	.75	[0.53, 1.58]	1.08	.79	[0.62, 1.90]	1.08	.78	[0.61, 1.92]
	High school	30	0.69	.18	[0.40, 1.19]	0.75	.34	[0.42, 1.34]	0.72	.30	[0.39, 1.33]
	Less than high school	14	0.62	.16	[0.32, 1.21]	0.73	.36	[0.37, 1.43]	0.60	.17	[0.29, 1.24]
Employment status	Employed	55	Reference			Reference			Reference		
	Unemployed	21	0.77	.33	[0.46, 1.30]	0.88	.62	[0.52, 1.48]	0.92	.75	[0.54, 1.56]
	Retired/disabled	24	1.34	.30	[0.77, 2.32]	1.30	.38	[0.72, 2.35]	1.20	.55	[0.66, 2.19]
Health-related social networks	Score of 0 (*no health-related networks*)	7				Reference			Reference		
	Score of 1	21				6.36	**<.001**	[3.09, 13.08]	6.94	**<.001**	[3.25, 14.83]
	Score of 2	32				6.02	**<.001**	[3.07, 11.78]	5.45	**<.001**	[2.66, 11.17]
	score of 3 (*strong health-related networks*)	41				8.72	**<.001**	[4.48, 16.96]	7.92	**<.001**	[3.89, 16.10]
Talked with doctors about health issues related to H1N1	No	86				Reference			Reference		
	Yes	14				3.16	**<.005**	[1.59, 6.28]	2.98	**<.005**	[1.41, 6.33]
Concerned about self or someone in your immediate family getting sick from H1N1 during the next year	Not much concerned	38							Reference		
	Little concerned	38							2.54	**<.001**	[1.62, 3.96]
	Somewhat concerned	16							3.03	**<.005**	[1.53, 5.97]
	Very concerned	8							6.08	**<.001**	[3.18, 11.62]
Knowledge about A(H1N1) virus transmission	Score of 0 (no correct answer)	10							Reference		
	Score of 1 (one correct answer)	45							1.78	.07	[0.95, 3.34]
	Score of 2 (two correct answers)	44							2.74	**<.005**	[1.41, 5.35]
*Began coughing with your mouth covered*			Tier-1: SEP			Tier-2: SEP and interpersonal networks			Tier-3: SEP, interpersonal networks, and intrapersonal factors		
Factors	Items	Percentage	aOR	*p*	95% CI	aOR	*p*	95% Cl	aOR	*p*	95% CI
Household Income	≤$14,999	14	Reference			Reference			Reference		
	$15,000-34,999	21	1.20	.49	[0.71, 2.02]	1.01	.98	[0.59, 1.73]	1.01	.97	[0.58, 1.76]
	$35,000-59,999	23	0.74	.28	[0.43, 1.27]	0.84	.54	[0.47, 1.48]	0.88	.68	[0.49, 1.58]
	≥$60,000	42	1.04	.89	[0.61, 1.79]	1.08	.79	[0.61, 1.92]	1.14	.65	[0.64, 2.04]
Education	Bachelor’s degree or higher	28	Reference			Reference			Reference		
	Some college	29	1.40	.24	[0.80, 2.45]	1.61	.13	[0.87, 3.00]	1.57	.15	[0.85, 2.92]
	High school	30	1.48	.18	[0.83, 2.61]	1.70	.10	[0.91, 3.17]	1.80	.08	[0.94, 3.46]
	Less than high school	14	1.43	.30	[0.73, 2.82]	1.80	.09	[0.90, 3.60]	1.81	.11	[0.88, 3.72]
Employment status	Employed	55	Reference			Reference			Reference		
	Unemployed	21	0.75	.29	[0.45, 1.27]	0.83	.50	[0.49, 1.42]	0.82	.49	[0.47, 1.42]
	Retired/disabled	24	1.62	.07	[0.96, 2.75]	1.70	.06	[0.97, 2.98]	1.78	**.05**	[1.00, 3.16]
Health-related social networks	Score of 0 *(no health-related networks)*	7				Reference			Reference		
	Score of 1	21				2.24	**.05**	[1.02, 4.95]	2.02	.09	[0.89, 4.56]
	Score of 2	32				2.06	.07	[0.95, 4.44]	1.83	.13	[0.83, 4.03]
	Score of 3 (*strong health-related networks*)	41				3.14	**<.01**	[1.47, 6.72]	2.70	**.01**	[1.26, 5.82]
Talked with doctors about health issues related to H1N1	No	86				Reference			Reference		
	Yes	14				4.02	**<.001**	[2.39, 6.76]	3.39	**<.001**	[1.93, 5.97]
Concerned about self or someone in your immediate family getting sick from H1N1 during the next year	Not much concerned	38							Reference		
	Little concerned	38							1.51	.11	[0.91, 2.52]
	Somewhat concerned	16							1.64	.10	[0.92, 2.95]
	Very concerned	8							2.53	**<.01**	[1.28, 5.04]
Knowledge about A(H1N1) virus transmission	Score of 0 (no correct answer)	10							Reference		
	Score of 1 (one correct answer)	45							1.34	.40	[0.68, 2.65]
	Score of 2 (two correct answers)	44							2.11	**.04**	[1.04, 4.31]

*Note*. AOR = adjusted odds ratio; CI = confidence interval. Adjusted for age, gender, and race/ethnicity; bold values indicate statistical significance (*p* < .05).

### Social Distancing Measures (Table 3)

Our data showed that SEP exerted significant influence on preventative behaviors uptake; retired or disabled people were more likely to stay home or avoid crowded places compared with employed persons, but the unemployed were less likely to do so. Those with higher annual incomes were less likely to stay home during the A(H1N1) pandemic. Social distancing behaviors had similar predictors to hygienic practices, specifically, having A(H1N1)-related concern and knowledge, a health-related social network, and discussing A(H1N1) with a doctor. Compared with those less concerned about themselves or loved ones being infected with A(H1N1), those who were very concerned were almost seven times more likely to have avoided places where people gathered, 4.24 times more likely to have reduced human contact, and 3.36 times more likely to have stayed home. Having higher levels of knowledge about A(H1N1)’s mode of transmission had a significant impact on behavior. People with knowledge scores of 2 were 2.91 times more likely to have avoided crowded places, 7.26 times more likely to have reduced human contact, and 2.45 times more likely to have stayed home, compared to those with no knowledge.

**Table 3.** Hierarchical Logistic Regression Analyses Examining the Association Between *Social Distancing Behaviors* and (a) Socioeconomic Position (SEP); (b) SEP and Interpersonal Networks: Health-Related Social Networks and Health Information-Seeking Behaviors; (c) SEP, Interpersonal Networks, and Intrapersonal Factors: A(H1N1)-Related Concern and Knowledge.

*Avoided places where many people are gathered together, like sporting events, malls, or public transportation*			Tier 1: SEP			Tier 2: SEP and interpersonal networks			Tier 3: SEP, interpersonal networks, and intrapersonal factors		
Factors	Items	Percentage	aOR	*p*	95% CI	aOR	*p*	95% CI	aOR	*p*	95% CI
Household income	≤$14,999	14	Reference			Reference			Reference		
	$15,000-$34,999	21	1.30	.38	[0.72, 2.35]	1.16	.63	[0.63, 2.14]	0.95	.86	[0.53, 1.70]
	$35,000-$59,999	23	0.40	**<.01**	[0.21, 0.76]	0.43	**.02**	[0.21, 0.85]	0.41	**<.01**	[0.21, 0.78]
	≥$60,000	42	0.66	.22	[0.34, 1.28]	0.67	.25	[0.33, 1.33]	0.67	.22	[0.35, 1.27]
Education	Bachelor’s degree or higher	28	Reference			Reference			Reference		
	Some college	29	0.90	.75	[0.47, 1.71]	1.08	.82	[0.55, 2.13]	1.03	.94	[0.52, 2.05]
	High school	30	0.70	.26	[0.38, 1.30]	0.77	.43	[0.41, 1.47]	0.64	.18	[0.34, 1.23]
	Less than high school	14	1.32	.46	[0.62, 2.81]	1.61	.23	[0.74, 3.52]	1.29	.51	[0.61, 2.75]
Employment status	Employed	55	Reference			Reference			Reference		
	Unemployed	21	0.74	.35	[0.40, 1.39]	0.83	.55	[0.44, 1.55]	0.80	.50	[0.42, 1.52]
	Retired/disabled	24	1.85	**.04**	[1.03, 3.32]	**1.88**	**.05**	[1.01, 3.48]	1.87	**.05**	[1.01, 3.45]
Health-related social networks	Score of 0 (*no health-related networks*)	7				Reference			Reference		
	Score of 1	21				2.68	**.02**	[1.20, 5.97]	2.58	**.03**	[1.10, 6.01]
	Score of 2	32				2.12	**.05**	[0.99, 4.52]	1.55	.28	[0.69, 3.48]
	Score of 3 (*strong health-related networks*)	41				2.94	**<.01**	[1.36, 6.36]	2.67	**.02**	[1.17, 6.08]
Talked with doctors about health issues related to H1N1	No	86				Reference			Reference		
	Yes	14				3.49	**<.001**	[2.05, 5.95]	2.83	**<.001**	[1.62, 4.95]
Concerned about self or someone in your immediate family getting sick from H1N1 during the next year	Not much concerned	38							Reference		
	Little concerned	38							3.55	**<.001**	[1.98, 6.37]
	Somewhat concerned	16							6.27	**<.001**	[3.32, 11.83]
	Very concerned	8							6.88	**<.001**	[3.40, 13.92]
Knowledge about A(H1N1) virus transmission	Score of 0 (no correct answer)	10							Reference		
	Score of 1 (one correct answer)	45							2.39	**.03**	[1.11, 5.12]
	Score of 2 (two correct answers)	44							2.91	**<.01**	[1.31, 6.43]
*Reduced human contact with people outside of your immediate family such as signs of affection (hug/kiss), shaking hands or sign of peace during worship*			Tier 1: SEP			Tier 2: SEP and interpersonal networks			Tier 3: SEP, interpersonal networks, and intrapersonal factors		
Factors	Items	Percentage	aOR	*p*	95% CI	aOR	*p*	95% CI	aOR	*p*	95% CI
Household income	≤$14,999	14	Reference			Reference			Reference		
	$15,000-$34,999	21	1.40	.26	[0.77, 2.54]	1.25	.48	[0.67, 2.34]	1.22	.53	[0.66, 2.26]
	$35,000-$59,999	23	0.64	.18	[0.33, 1.23]	0.71	.34	[0.35, 1.42]	0.72	.36	[0.36, 1.45]
	≥$60,000	42	0.98	.95	[0.50, 1.90]	0.99	.98	[0.49, 2.00]	1.06	.87	[0.54, 2.06]
Education	Bachelor’s degree or higher	28	Reference			Reference			Reference		
	Some college	29	1.04	.91	[0.56, 1.92]	1.30	.44	[0.67, 2.50]	1.24	.51	[0.65, 2.39]
	High school	30	0.86	.63	[0.47, 1.59]	0.97	.93	[0.51, 1.84]	1.03	.93	[0.54, 1.98]
	Less than high school	14	1.46	.33	[0.68, 3.13]	1.85	.14	[0.82, 4.15]	1.73	.16	[0.80, 3.73]
Employment Status	Employed	55	Reference			Reference			Reference		
	Unemployed	21	1.01	.97	[0.55, 1.86]	1.14	.68	[0.62, 2.09]	1.20	.57	[0.63, 2.31]
	Retired/disabled	24	1.57	.14	[0.86, 2.84]	1.57	.16	[0.84, 2.91]	1.64	.12	[0.88, 3.07]
Health-related Social Networks	Score of 0 (*no health-related networks*)	7				Reference			Reference		
	Score of 1	21				2.25	.07	[0.95, 5.35]	1.64	.29	[0.65, 4.11]
	Score of 2	32				2.24	**.04**	[1.04, 4.83]	1.67	.23	[0.72, 3.87]
	Score of 3 (*strong health-related networks*)	41				3.44	**<.01**	[1.57, 7.57]	2.71	**.02**	[1.16, 6.29]
Talked with doctors about health issues related to H1N1	No	86				Reference			Reference		
	Yes	14				3.37	**<.001**	[2.01, 5.66]	2.75	**<.001**	[1.60, 4.74]
Concerned about self or someone in your immediate family getting sick from H1N1 during the next year	Not much concerned	38							Reference		
	Little concerned	38							1.39	.29	[0.76, 2.53]
	Somewhat concerned	16							3.62	**<.001**	[1.89, 6.92]
	Very concerned	8							4.24	**<.001**	[2.12, 8.47]
Knowledge about A(H1N1) virus transmission	Score of 0 (no correct answer)	10							Reference		
	Score of 1 (one correct answer)	45							4.34	**<.01**	[1.76, 10.69]
	Score of 2 (two correct answers)	44							7.26	**<.001**	[2.91, 18.08]
*Stayed home*			Tier 1: SEP			Tier 2: SEP and interpersonal networks			Tier 3: SEP, interpersonal networks and intrapersonal factors		
Factors	Items	Percentage	aOR	*p*	95% CI	aOR	*p*	95% CI	aOR	*p*	[95% CI
Household income	≤$14,999	14	Reference			Reference			Reference		
	$15,000-$34,999	21	0.85	.62	[0.43, 1.64]	0.78	.48	[0.39, 1.56]	0.65	.24	[0.31, 1.33]
	$35,000-$59,999	23	0.46	**.05**	[0.21, 0.99]	0.45	**.04**	[0.21, 0.97]	0.45	**.05**	[0.20, 1.00]
	$60,000	42	0.31	**<.01**	[0.14, 0.68]	0.28	**<.01**	[0.13, 0.63]	0.25	**<.01**	[0.12, 0.55]
Education	Bachelor’s degree or higher	28	Reference			Reference			Reference		
	Some college	29	1.10	.87	[0.36, 3.38]	1.17	.80	[0.36, 3.77]	1.22	.74	[0.37, 4.05]
	High school	30	2.44	.08	[0.91, 6.58]	2.64	.06	[0.96, 7.25]	2.56	.08	[0.89, 7.41]
	Less than high school	14	1.68	.29	[0.64, 4.40]	1.84	.23	[0.69, 4.90]	1.87	.23	[0.68, 5.12]
Employment status	Employed	55	Reference			Reference			Reference		
	Unemployed	21	0.48	**.02**	[0.26, 0.88]	0.46	**.01**	[0.25, 0.85]	0.51	**.04**	[0.27, 0.97]
	Retired/disabled	24	2.35	**<.01**	[1.35, 4.07]	2.23	**<.01**	[1.26, 3.96]	2.00	**.03**	[1.07, 3.72]
Health-related social networks	Score of 0 (*no health-related networks*)	7				Reference			Reference		
	Score of 1	21				1.14	.75	[0.49, 2.67]	1.13	.77	[0.49, 2.59]
	Score of 2	32				1.64	.27	[0.68, 3.96]	1.16	.75	[0.46, 2.91]
	Score of 3 (*strong health-related networks*)	41				1.60	.24	[0.73, 3.53]	1.38	.43	[0.62, 3.09]
Talked with doctors about health issues related to H1N1	No	86				Reference			Reference		
	Yes	14				1.66	.10	[0.90, 3.07]	1.52	.19	[0.82, 2.81]
Concerned about self or someone in your immediate family getting sick from H1N1 during the next year	Not much concerned	38							Reference		
	Little concerned	38							2.44	**.03**	[1.08, 5.53]
	Somewhat concerned	16							2.42	.06	[0.97, 6.03]
	Very concerned	8							3.36	**<.01**	[1.49, 7.58]
Knowledge about A(H1N1) virus transmission	Score of 0 (no correct answer)	10							Reference		
	Score of 1 (one correct answer)	45							1.53	.24	[0.75, 3.14]
	Score of 2 (two correct answers)	44							2.45	**.02**	[1.17, 5.13]

*Note*. AOR = adjusted odds ratio; CI = confidence interval. Adjusted for age, gender, and race/ethnicity; bold values indicate statistical significance (*p* < .05).

### Vaccine Acceptance (Table 4)

Those somewhat (AOR = 2.12) or very concerned (AOR = 2.84) about A(H1N1) and those who discussed health issues related to A(H1N1) with doctors (AOR = 3.33) were much more likely to seek vaccination for themselves or loved ones, compared with their counterparts. The unemployed disproportionately sought vaccines during the A(H1N1) pandemic.

**Table 4.** Hierarchical Logistic Regression Analyses Examining the Association Between *Seeking Out a Vaccine for Self and Loved Ones* and (a) Socioeconomic Position (SEP); (b) SEP and Interpersonal Networks: Health-Related Social Networks and Health Information-Seeking Behaviors; (c) SEP, Interpersonal Networks, and Intrapersonal Factors: A(H1N1)-Related Concern and Knowledge.

*Sought out a vaccine for you or your loved ones*			Tier-1: SEP			Tier-2: SEP and interpersonal networks			Tier-3: SEP, interpersonal networks and intrapersonal factors		
Factors	Items	Percentage	aOR	*p*	95% Cl	aOR	*p*	95% Cl	aOR	*p*	95% Cl
Household income	≤$14,999	14	Reference			Reference			Reference		
	$15,000-$34,999	21	0.81	.46	[0.46, 1.4]	0.68	.21	[0.38, 1.24]	0.75	.39	[0.38, 1.46]
	$35,000-$59,999	23	0.97	.93	[0.53, 1.79]	1.17	.65	[0.60, 2.26]	1.24	.55	[0.61. 2.56]
	≥$60,000	42	1.48	.17	[0.84, 2.61]	1.64	.12	[0.88, 3.04]	1.84	.09	[0.92. 3.69]
Education	Bachelor’s degree or higher	28	Reference			Reference			Reference		
	Some college	29	1.37	.26	[0.79, 2.37]	1.68	.08	[0.94, 2.98]	1.72	.06	[0.97, 3.04]
	High school	30	1.08	.80	[0.59, 1.99]	1.32	.38	[0.70, 2.49]	1.25	.49	[0.66, 2.36]
	Less than high school	14	1.33	.42	[0.66, 2.68]	1.78	.13	[0.84, 3.78]	1.52	.29	[0.70, 3.30]
Employment status	Employed	55	Reference			Reference			Reference		
	Unemployed	21	1.72	**.05**	[1.01, 2.93]	1.87	**.03**	[1.08, 3.24]	2.09	**.01**	[1.19, 3.65]
	Retired/disabled	24	1.17	.60	[0.65, 2.11]	1.10	.77	[0.59, 2.02]	1.07	.83	[0.58, 1.97]
Health-related social networks	Score of 0 (*no health-related networks*)	7				Reference			Reference		
	Score of 1	21				0.82	.74	[0.27, 2.52]	0.88	.81	[0.32, 2.42]
	Score of 2	32				1.19	.75	[0.42, 3.34]	1.22	.69	[0.46, 3.22]
	Score of 3 (*strong health-related networks*)	41				1.55	.39	[0.57, 4.26]	1.64	.30	[0.65, 4.15]
Talked with doctors about health issues related to H1N1	No	86				Reference			Reference		
	Yes	14				3.48	**<.001**	[2.11, 5.75]	3.33	**<.001**	[1.97, 5.62]
Concerned about self or someone in your immediate family getting sick from H1N1 during the next year	Not much concerned	38							Reference		
	Little concerned	38							1.06	.84	[0.61, 1.83]
	Somewhat concerned	16							2.12	**.03**	[1.09, 4.15]
	Very concerned	8							2.84	**.02**	[1.17. 6.92]
Knowledge about A(H1N1) virus transmission	Score of 0 (no correct answer)	10							Reference		
	Score of 1 (one correct answer)	45							0.53	.15	[0.23, 1.26]
	Score of 2 (two correct answers)	44							0.78	.57	[0.34, 1.82]

*Note.* AOR = adjusted odds ratio; CI = confidence interval. Adjusted for age, gender, and race/ethnicity; bold values indicate statistical significance (*p* < .05).

## Discussion

This study uniquely contributes to the fields of emergency risk communication and PHEP and response by investigating five sets of independent factors drawing from the literature on social determinants, health communication and health behavior theories. We specifically examined how SEP, health-related social networks and consultations, and threat-related concern and knowledge are associated with behavioral responses during a pandemic. Our results show each NPI had a distinct set of predictors—both intrapersonal and interpersonal—which combined to influence PHEP outcomes. Across behaviors, higher levels of pandemic-related concern and higher scores on social (interpersonal communication) networks were positively associated with preventive behavior uptake.

SEP and intrapersonal (affective and cognitive) factors, such as concern and knowledge, have remained as significant predictors for the uptake of numerous behaviors, including hygiene and social distancing behaviors. This suggests an opportunity to focus pandemic messages on modifiable factors, creating campaigns, which focus on knowledge and risk perception to increase public uptake of behaviors. This is promising, as the impact of social determinants, such as social class, on PHEP outcomes could be blunted with communications through social networks and providers by increasing risk perceptions and knowledge. However, it is critical to note that heightened risk perceptions should be in concomitance with ways to address any fear that public communications might engender.

Beyond intrapersonal factors, our data showed doctors’ opinions strongly affect health behaviors, identifying providers as important sources of pandemic-related messages. However, lower SEP individuals may face challenges accessing health care or health consultations, and thus, social class may still play a significant role in such interventions. Health-related social networks were found as important predictors of hygiene- and social distancing–based strategies, prompting further study into their ability to quickly transmit preventive information during a pandemic’s onset, particularly among groups with less access to health care.

Despite the importance of modifiable intra- and interpersonal factors, SEP remains highly relevant. The SIM, for example, posits that the ability to act on pandemic information may be differentially associated with SEP factors, as evidenced by income and employment status’ influence on social distancing behaviors. Restricting movement outside the home may be difficult to act on given a range of factors, including career or family needs (Blake, Blendon, & Viswanath, 2010). Staying home during pandemics is often economically untenable for those concerned about losing their job due to absence, or who cannot afford missed days; lower wage occupations often have less flexible scheduling (Blake et al., 2010; Blumenshine et al., 2008). Our study found inconsistent relationships between SEP and several prevention behavior outcomes. For example, higher income earners were less likely to stay home. These inconsistencies might be explained at the structural level. The A(H1N1) pandemic occurred during the economic crisis, with the U.S. suffering high unemployment rates of 9.8% to 10.2% (Goodman, 2009; U.S. Bureau of Labor Statistics, 2010). Thus, even relatively high earners may have felt pressured to work due to understafﬁng or from fear of downsizing. Message strategies that take these challenges into account may encourage employers to adapt sick leave and work-from-home policies during pandemics.

### Public Health Implications

Our study found different factors working together contributed to an individual’s intention to perform NPIs and seek vaccines. Although SEP played a role in many behaviors, modifiable factors, such as concern and knowledge, were consistent predictors. Additionally, the presence of communication channels, including providers and health-related networks, emerged as important factors for NPIs uptake. Public health educators are cautioned to consider the context of each behavior when crafting messages, particularly for behaviors placing greater burdens on participants’ resources, for example, social distancing. Care must be taken to deliver messages that are both easy to understand and that properly contextualize risk. Per the SIM Model, while social determinants often play a significant role in public health outcomes, these findings show that strategic communications through interpersonal communication networks to raise risk perceptions and knowledge can overcome structural barriers.

### Limitations

The cross-sectional study design limited us from drawing causal relationships among SEP, people’s concern and knowledge, health information seeking, and behavior outcomes. This study was conducted in late February and early March 2010, when CDC’s emergency risk communication campaigns on precautionary methods against H1N1 (beginning April, 2009; CDC, 2010c) and vaccine programs targeting priority groups (announced in August, 2009; CDC, 2009) had already provided nation-wide coverage of H1N1-related knowledge and prevention methods and when pandemic uncertainties had passed their peak. It is likely our data did not capture the total effect social disparities exerted in the pandemic’s initial phases. Therefore, rapid survey tools are needed to gather timely information during future public health emergencies. Measures assessing individual’s social networks for obtaining and sharing health information are limited, and what types of information are transmitted through social networks remains unknown. Techniques analyzing information exchange on social media and assessing the information environment people are exposed to in real time will assist in designing future communication strategies.

### Conclusions

Our findings indicate that modifiable conditions, such as concern and knowledge, may be fertile territory for communication campaigns, but that SEP-related factors may inhibit certain preventative behaviors, particularly social distancing. Emergency risk communication may benefit from leveraging information exchange within health-related social networks and with providers, as these notably influenced a range of PHEP behaviors. When crafting communications, messages should be easily actionable and understandable, and take the context of the target audience into account, so they do not perpetuate communication inequalities.
